# Expression of cAMP and oxidative phosphorylation‐related lncRNAs in non‐functioning pituitary adenomas

**DOI:** 10.1111/jcmm.18011

**Published:** 2023-11-06

**Authors:** Mohammad Taheri, Amir Nicknam, Atena Bagan, Solat Eslami, Azadeh Rakhshan, Soudeh Ghafouri‐Fard

**Affiliations:** ^1^ Institute of Human Genetics Jena University Hospital Jena Germany; ^2^ Urology and Nephrology Research Center Shahid Beheshti University of Medical Sciences Tehran Iran; ^3^ Phytochemistry Research Center Shahid Beheshti University of Medical Sciences Tehran Iran; ^4^ Department of Medical Biotechnology, School of Medicine Alborz University of Medical Sciences Karaj Iran; ^5^ Dietary Supplements and Probiotic Research Center Alborz University of Medical Sciences Karaj Iran; ^6^ Department of Pathology, Shohada‐e Tajrish Hospital Shahid Beheshti University of Medical Sciences Tehran Iran; ^7^ Men's Health and Reproductive Health Research Center Shahid Beheshti University of Medical Sciences Tehran Iran

**Keywords:** cAMP, lncRNA, non‐functioning pituitary adenomas, oxidative phosphorylation

## Abstract

Non‐functioning pituitary adenomas (NFPAs) are benign lesions in the pituitary gland with important morbidities. In this study, based on a bioinformatics analysis to identify the genes and pathways that display significant differences between tumour tissues of NFPA patients and normal pituitary tissues, we selected lncRNAs related to cAMP and oxidative phosphorylation pathways, namely DNAH17‐AS1, LINC00706 and SLC25A5‐AS1. Then, we aimed to investigate by means of RT‐qPCR, the expression of these lncRNAs along with two other lncRNAs, namely CADM3‐AS1 and MIR7‐3HG in NFPA samples compared to that in healthy tissues adjacent to the tumours. Transcripts of DNAH17‐AS1, LINC00706 and MIR7‐3HG were lower in NFPA samples compared with controls (Expression ratios (95% CI) = 0.43 (0.23–0.78), 0.58 (0.35–0.96) and 0.58 (0.35–0.96); *p*‐values = 0.009, 0.025 and 0.036, respectively). AUC values of ROC curves of DNAH17‐AS1, LINC00706 and MIR7‐3HG were 0.62, 0.61 and 0.62, respectively. Expression of CADM3‐AS1 was associated with the gender of patients in a way that it was lower in female patients (*p*‐value = 0.04). The level of SLC25A5‐AS1 was lower in subjects with disease duration lower than 1 year (*p*‐value = 0.048). We showed dysregulation of three lncRNAs in NFPA tissues and potentiates these lncRNAs as important regulators of pathogenic events in these tumours.

## 1 INTRODUCTION

Non‐functioning pituitary adenomas (NFPAs) are benign lesions of pituitary gland originating from adenohypophysis cells. Except for mild symptoms in some cases, they do not have clinical or biochemical signs of excessive hormone secretion.^1^ For this reason, they often remain undiagnosed until they are either discovered incidentally or grow into macroadenomas, which cause compression on the chiasm, leading to headache and visual field defect which are sometimes irreversible.^2^ Surgery is definitely indicated for tumour syndrome, but there is still debate about other aspects of NFPA, including hormonal evaluation, follow‐up (especially post‐operative follow‐up) and management of post‐operative recurrence.^3^

While in the case of hormone‐secreting tumours, disease monitoring is possible by measuring pituitary hormones, the only option for clinical follow‐up in non‐functioning tumours is MRI. Hence, identifying non‐invasive biomarkers for non‐functioning tumours is clinically useful for early diagnosis and follow‐up.^4^ The evaluation of a perfect tumour biomarker should be easy, consistent, cost‐effective and performable with a method that has high sensitivity and specificity. Such a biomarker should also be specific to a certain disease, be capable to distinguish between different physiological states being reliable among different ethnicities and genders.^5^

Non‐coding RNAs have many of these features, so they are often indicated as promising biomarkers. Non‐coding RNAs are involved in many cellular processes and are divided into two groups: small non‐coding RNAs (less than 200 base pairs in length) and long non‐coding RNAs or lncRNA (with a length of more than 200 bp).^6^ In the pathobiology of cancer, thousands of intracellular factors are altered, including signalling pathways, energy metabolism, gene transcription regulators, etc.^7^, ^8^, ^9^, ^10^ Signalling pathways are often stimulated by signalling molecules found on cell membranes or within the cytoplasm, such as growth factors, hormones, or ions. For signal transduction, a variety of intracellular chemicals known as secondary messengers are used, an important one of which is cyclic adenosine monophosphate (cAMP).^11^ Apart from this, the observation that glycolysis is augmented in cancer cells compared to normal cells leads to the hypothesis that oxidative phosphorylation is generally reduced in cancer, which is true for many malignancies, but in some cancers including leukaemia and lymphoma, where mitochondrial metabolism is not disturbed, this assumption is challenged.^12^

We performed a bioinformatics analysis based on a previously conducted microarray experiment to identify the genes and pathways that display significant differences between tumour tissues of NFPA patients and normal pituitary tissusses of healthy individuals.^13^ We aimed to investigate by means of RT‐qPCR, the expression of DNAH17‐AS1, LINC00706, SLC25A5‐AS1, CADM3‐AS1 and MIR7‐3HG in NFPA samples compared to that in healthy tissues adjacent to the tumours.

Based on the information above, our study will yield more comprehensive knowledge about the role of the aforementioned pathways and molecules in NFPA, which will have the potential to be used for better management of the disease.

## 2 MATERIALS AND METHODS

### 2.1 Patients

Expression of DNAH17‐AS1, LINC00706, SLC25A5‐AS1, CADM3‐AS1 and MIR7‐3HG lncRNAs was assessed in 46 pairs of NFPA specimens and paired non‐cancerous specimens. Tissue samples were obtained during tumour excision from patients admitted to Loghman and Nikan hospitals during 2021–2022. Patients took no chemo/radiotherapy prior to lesion excision. The study protocol was approved by the ethical committee of Shahid Beheshti University of Medical Sciences. Informed consent forms were obtained from all participants.

### 2.2 Experiments

Total RNA was extracted using RNJia extraction kit (Roje Technologies Company). Afterwards, RNA was converted to cDNA using AddScript cDNA Synthesis Kit (ADDBIO Company). Expression of DNAH17‐AS1, LINC00706, SLC25A5‐AS1, CADM3‐AS1 and MIR7‐3HG was determined using RealQ Plus 2x Master Mix Green with high ROX (AMPLIQON, Denmark), and primers were synthesized by the METABION Company. Primers are shown in Table 1.

**TABLE 1:** Information about primers and amplicons.

Name	Type	Sequence	Primer length	PCR product length
DNAH17‐AS1‐F	lncRNA	GAACGCAAAGAAACTCGCCC	20	169
DNAH17‐AS1‐R		GCTGGACCGAATGTGCTCTA	20	
SLC25A5‐AS1‐F	lncRNA	AGGGCTTTATTTGGAGAGAGGT	22	139
SLC25A5‐AS1‐R		AGTGATGGCGAGGTGTATCG	20	
LINC00706‐F	lncRNA	GCCACAAACAGAATCCAAGAC	21	143
LINC00706‐R		CCAAAAGACACTCCCCCAAC	20	
MIR7‐3HG‐F	lncRNA	AGGTCCGAGAAGAGAACCGC	20	156
MIR7‐3HG‐R		GAAGGCAGGGTCAGTTGTGG	20	
CADM3‐AS1‐F	lncRNA	AGAGTAAGGAGGAGACCAGGA	21	169
CADM3‐AS1‐R		CGTGTGGGGAGGTATTCATTG	21	
B2M‐F	mRNA	AGATGAGTATGCCTGCCGTG	20	105
B2M‐R		GCGGCATCTTCAAACCTCCA	20	

### 2.3 Statistical analysis

Statistical analyses and graphics were performed using SPSS v.22.0 (SPSS Inc.) and GraphPad Prism version 9.0 (GraphPad Software), respectively. Expression levels of DNAH17‐AS1, LINC00706, SLC25A5‐AS1, CADM3‐AS1 and MIR7‐3HG were compared between NFPA samples and adjacent non‐cancerous tissues using the Efficiency adjusted method. The normal/gaussian distribution of the values was judged by the Shapiro–wilk test. Wilcoxon matched‐pairs signed rank test or paired t‐test was used to recognize differentially expressed genes between two sets of samples.

The correlation between expressions of mentioned genes was evaluated using the Spearman correlation coefficient. Expression levels of genes were compared between different subgroups using the Mann–Whitney and Kruskal–Wallis one‐way anova test. The chi‐square test was used to find out the association between clinical parameters and gene expression levels.

## 3 RESULTS

### 3.1 General information.

Clinical data of patients are summarized in Table S1. Table 2 displays location and other characteristics of studied genes.

**TABLE 2:** General features of studied genes.

Name/Gene ID	Accession number	Location	Official full name	Gene type
DNAH17‐AS1	NR_102401.1	17q25.3	DNAH17 antisense RNA 1	ncRNA
SLC25A5‐AS1	NR_028443.1; NR_134914.1; NR_134915.1	Xq24	SLC25A5 antisense RNA 1	ncRNA
CADM3‐AS1	NR_037870.1	1q23.2	CADM3 antisense RNA 1	ncRNA
MIR7‐3HG	NR_027148.1	19p13.3	MIR7‐3 host gene	ncRNA
LINC00706	NR_108074.1	10p14	Long intergenic non‐protein coding RNA 706	ncRNA

### 3.2 Experiments

**FIGURE 1:**
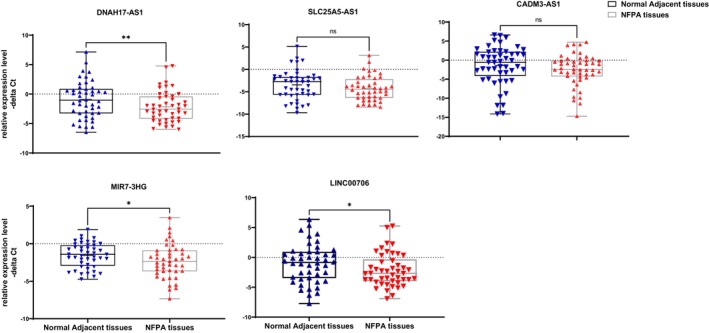
Levels of lncRNAs expression in non‐functional pituitary adenoma (NFPA) tissues compared with adjacent non‐tumoral tissues as described by –delta Ct values. Delta Ct Data were plotted as box‐and‐whisker plots showing median [line], mean [cross], interquartile range [box] and minimum and maximum values. Data were analysed using the Wilcoxon rank‐sum test or paired *t*‐test, and *p* < 0.05 was considered statistically significant. Asterisks designate significant differences between groups (*p‐value < 0.05, **p‐value < 0.01 ns; non‐significant).

Significant differences were detected in expression levels of DNAH17‐AS1, LINC00706 and MIR7‐3HG between NFPA tissues and adjacent tissues (Figure 1).

Table 3 shows the details of expression assays. Expression levels of DNAH17‐AS1, LINC00706 and MIR7‐3HG were lower in NFPA samples compared with controls (Expression ratios (95% CI) = 0.43 (0.23–0.78), 0.58 (0.35–0.96) and 0.58 (0.35–0.96); *p*‐values = 0.009, 0.025 and 0.036, respectively).

**TABLE 3:** The results of expression study of five lncRNAs in non‐functional pituitary adenoma (NFPA) tissues compared with the neighbouring normal tissues.

Studied genes	Expression ratio (95% CI)	SEM	*p*‐Value
DNAH17‐AS1	0.43 (0.23–0.78)	0.42	**0.009**
SLC25A5‐AS1	0.58 (0.3–1.1)	0.45	0.09
CADM3‐AS1	0.44 (0.15–1.33)	0.78	0.15
MIR7‐3HG	0.58 (0.35–0.96)	0.52	**0.036**
LINC00706	0.58 (0.35–0.96)	0.36	**0.025**

*Note*: Bold indicates *p* < 0.05.

**FIGURE 2:**
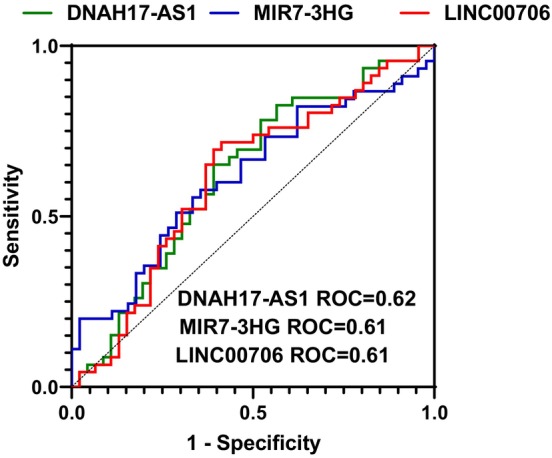
ROC curves of DNAH17‐AS1, MIR7‐3HG and LINC00706 lncRNAs for discrimination of NFPA tumours from adjacent normal tissues. AUC designates an area under the ROC curve.

Since we detected a significant difference in the expression of DNAH17‐AS1, LINC00706 and MIR7‐3HG between tumoral and non‐tumoral tissues, we depicted ROC curves for these lncRNAs to show their diagnostic value (Figure 2).

Detailed information about ROC curves of DNAH17‐AS1, MIR7‐3HG and LINC00706 lncRNA is shown in Table 4. AUC values of DNAH17‐AS1, LINC00706 and MIR7‐3HG were 0.62, 0.61 and 0.62, respectively.

**TABLE 4:** ROC curve analysis of lncRNAs transcript levels for discrimination of NFPA tumours genes from adjacent non‐tumoral tissues.

DNAH17‐AS1				SLC25A5‐AS1				CADM3‐AS1				MIR7‐3HG				LINC00706			
AUC ± SD	Sensitivity	Specificity	*p*‐Value	AUC ± SD	Sensitivity	Specificity	*p*‐Value	AUC ± SD	Sensitivity	Specificity	*p*‐Value	AUC ± SD	Sensitivity	Specificity	*p*‐Value	AUC ± SD	Sensitivity	Specificity	*p*‐Value
0.62 ± 0.05	0.65	0.6	0.04	0.57 ± 0.06	0.75	0.52	0.26	0.6 ± 0.05	0.84	0.43	0.09	0.62 ± 0.06	0.56	0.67	0.05	0.61 ± 0.06	0.69	0.61	0.05

Expression levels of LINC00706 were significantly correlated with all other lncRNAs except for MIR7‐3HG in both NFPA tissues and adjacent tissues (Table 5). Moreover, expression levels of CADM3‐AS1 were correlated with those of DNAH17‐AS1 and SLC25A5‐AS1 in both sets of tissues.

**TABLE 5:** Spearman's correlations between lncRNAs expression levels among tumour tissues (*N* = 46) and adjacent non‐tumoral tissues (*N* = 46).

	SLC25A5‐AS1		CADM3‐AS1		MIR7‐3HG		LINC00706	
	Non‐tumoral tissue	Tumour	Non‐tumoral tissue	Tumour	Non‐tumoral tissue	Tumour	Non‐tumoral tissue	Tumour
DNAH17‐AS1	0.91**	0.86**	0.82**	0.79**	0.11	0.14	0.96**	0.91**
SLC25A5‐AS1			0.76**	0.74**	0.29*	0.13	0.92**	0.89**
CADM3‐AS1					0.19	0.09	0.79**	0.76**
MIR7‐3HG							0.22	0.18

*Note*: **p < 0.001.

Finally, we assessed association between expression levels of DNAH17‐AS1, LINC00706, SLC25A5‐AS1, CADM3‐AS1 and MIR7‐3HG and clinicopathological data (Table 6). Expression of CADM3‐AS1 was associated with gender of patients in a way that it was lower in female patients (*p*‐value = 0.04). Expression of SLC25A5‐AS1 was lower in subjects with disease duration lower than 1 year (*p*‐value = 0.048).

**TABLE 6:** Comparison of expression levels of lncRNAs in NFPA patients with different clinical features. Mann–Whitney and Kruskal–Wallis one‐way anova tests were used for comparing gene expression levels.

Parameters	Subclasses	Number of patients (%)	Expression levels of DNAH17‐AS1 (mean ± SD)	*p*‐Value	Expression levels of SLC25A5‐AS1 (mean ± SD)	*p*‐Value	Expression levels of CADM3‐AS1 (mean ± SD)	*p*‐Value	Expression levels of MIR7‐3HG (mean ± SD)	*p*‐Value	Expression levels of LINC00706 (mean ± SD)	*p*‐Value
Tumour subtypes	NFPA	36	−1.85 ± 0.46	0.46	−4.17 ± 0.46	0.85	−2.17 ± 0.7	0.35	−2.32 ± 0.36	0.91	−1.98 ± 0.49	0.91
	NFPA +CD + AP	11	−2.5 ± 0.75		−4.1 ± 0.69		−3.2 ± 1		−2.7 ± 0.72		−1.92 ± 0.69	
Age	22–48	24	−1.84 ± 0.53	0.68	−3.9 ± 0.53	0.48	−1.95 ± 0.75	0.38	−2.48 ± 0.41	0.96	−1.76 ± 0.53	0.58
	49–77	23	−2.19 ± 0.59		−4.4 ± 0.57		−2.9 ± 0.92		−2.35 ± 0.51		−2.19 ± 0.63	
Gender	Female	10	−2.89 ± 0.62	0.37	−4.46 ± 0.59	0.94	−4.15 ± 0.99	**0.04**	−1.31 ± 0.77	0.1	−3.13 ± 0.75	0.28
	Male	37	−1.78 ± 0.46		−4.06 ± 0.47		−1.95 ± 0.68		−2.72 ± 0.35		−1.66 ± 0.47	
Disease duration	<1y	24	−2.4 ± 0.53	0.16	−4.92 ± 0.48	**0.048**	−3.08 ± 0.74	0.085	−2.24 ± 0.48	0.58	−2.57 ± 0.54	0.16
	> = 1 y	23	−1.56 ± 0.58		−3.34 ± 0.57		−1.72 ± 0.91		−2.61 ± 0.44		−1.35 ± 0.59	
Tumour Size (cm)	<500 mm^2^	16	−2.16 ± 0.66	0.66	−4.49 ± 0.64	0.77	−3.5 ± 1.28	0.62	−2.77 ± 0.53	0.51	−2.13 ± 0.63	0.41
	500–800 mm^2^	15	−2.46 ± 0.64		−4.38 ± 0.65		−1.96 ± 0.68		−1.77 ± 0.56		−2.64 ± 0.75	
	>800 mm^2^	16	−1.44 ± 0.74		−3.6 ± 0.73		−1.7 ± 0.97		−2.67 ± 0.6		−1.19 ± 0.73	
CSF leak	No	25	−2.3 ± 0.53	0.59	−4.64 ± 0.47	0.33	−2.54 ± 0.69	0.6	−2.25 ± 0.43	0.43	−2.3 ± 0.54	0.62
	Low flow	11	−1.98 ± 0.86		−3.89 ± 0.87		−3.18 ± 1.6		−3.13 ± 0.76		−1.45 ± 0.83	
	High flow	11	−1.39 ± 0.85		−3.29 ± 0.9		−1.36 ± 1.2		−2.1 ± 0.64		−1.73 ± 0.97	
Knosp classification	1	11	−2.28 ± 0.49	0.07	−4.27 ± 0.65	0.46	−3.56 ± 1.34	0.53	−1.61 ± 0.67	0.46	−2.06 ± 0.56	0.12
	2	20	−2.87 ± 0.57		−4.66 ± 0.54		−2.54 ± 0.76		−2.66 ± 0.4		−2.8 ± 0.57	
	3	16	−0.76 ± 0.77		−3.43 ± 0.79		−1.48 ± 1.12		−2.68 ± 0.67		−0.87 ± 0.84	
Invasiveness	Invasive	8	−1.95 ± 0.46	0.87	−4.02 ± 0.44	0.66	−2.04 ± 0.63	0.11	−2.28 ± 0.32	0.23	−1.82 ± 0.46	0.64
	Non‐invasive	39	−2.32 ± 0.6		−4.7 ± 0.76		−4.25 ± 1.45		−3.08 ± 1.12		−2.7 ± 0.81	
Drug history	Yes	8	−2.11 ± 0.43	0.56	−4.17 ± 0.43	0.79	−2.46 ± 0.65	0.83	−2.52 ± 0.36	0.39	−2.17 ± 0.45	0.23
	No	39	−1.54 ± 1.05		−4.06 ± 0.9		−2.2 ± 1.46		−1.91 ± 0.7		−1 ± 0.89	

*Note*: Bold indicates *p* < 0.05.

There was a significant positive association between age and invasiveness of NFPA (*χ* ^2^ = 4.24, *p*‐value = 0.039) and drug history (*χ* ^2^ = 5.74, *p*‐value = 0.017). Moreover, there was a significant positive association between disease duration and CSF leak (*χ* ^2^ = 9.4, *p*‐value = 0.043). Furthermore, a significant positive association was detected between Knosp classification and invasiveness of NFPA (*χ* ^2^ = 7.28, *p*‐value = 0.026). Lastly, a significant positive association was reported between tumour size and Knosp classification (*χ* ^2^ = 14.4, *p*‐value = 0.006) and invasiveness of NFPA (*χ* ^2^ = 8.17, *p*‐value = 0.017).

## 4 DISCUSSION

Expression assays can lead to the identification of important regulators of cell proliferation, apoptosis and other features in each tissue since dysregulated genes are potentially involved in these processes. In the current study, we selected lncRNAs related to cAMP and oxidative phosphorylation pathways to assess their expression in NFPA and adjacent tissues. Expression levels of DNAH17‐AS1, LINC00706 and MIR7‐3HG were lower in NFPA samples compared with controls. DNAH17‐AS1 has been shown to promote tumorigenesis and metastatic ability of non‐small cell lung cancer (NSCLC) cells through regulation of miR‐877‐5p/CCNA2 pathway.^14^ Another miRNA that regulates expression of CCNA2, namely miR‐130b has been shown to be dysregulated in pituitary adenomas.^15^ In the pancreatic cancer cells, DNAH17‐AS1 upregulation designates aggressive behaviour of tumour. When it is silenced in these cells, cell apoptosis is induced and viability, invasion and migration are decreased.^16^ It acts as a miR‐432‐5p sponge.^16^ Forthcoming studies are needed to find correlation between DNAH17‐AS1, other miRNAs and CCNA2 in this type of tumour.

MIR7‐3HG is regarded as a MYC‐dependent regulator of cell proliferation that suppresses autophagy through a regulatory loop that involves AMBRA1.^17^ AMBRA1 has been shown to be among genes associated with recurrence in lactotroph lesions based on a CGH array study.^18^ However, relation of this gene with NFPA has not been evaluated yet.

AUC values of ROC curves of DNAH17‐AS1, LINC00706 and MIR7‐3HG were 0.62, 0.61 and 0.62, respectively. Hence, none of these lncRNAs can be considered as appropriate markers for differentiation of NFPA from adjacent tissues.

Expression of CADM3‐AS1 was associated with gender of patients in a way that it was lower in female patients. CADM3‐AS1 is an lncRNA which is associated with regulation of protein translation, targeting and localization. Expression of this lncRNA in patients with lung adenocarcinoma has been associated with overall survival.^19^

Expression of SLC25A5‐AS1 was lower in subjects with disease duration lower than 1 year. Downregulation of SLC25A5‐AS1 in gastric cancer cells can facilitate cell growth and inhibit apoptosis through miR‐19a‐3p/PTEN/PI3K/AKT axis.^20^ This pathway has a fundamental role in the pathogenesis of tumours of endocrine system.^21^ However, the importance of SLC25A5‐AS1 in the regulation of this pathway in NFPA needs to be elucidated.

Finally, the observed correlations between a number of lncRNAs in this study suggest the presence of a functional network between these lncRNAs.

In brief, we reported dysregulation of three lncRNAs in NFPA. The current study potentiates these lncRNAs as important regulators of pathogenic events in these tumours. We propose conduction of functional assays to elaborate the mechanism of dysregulation of these lncRNAs and the functional consequences of these events.

## AUTHOR CONTRIBUTIONS

**Mohammad Taheri:** Data curation (equal); writing – original draft (equal). **Amir Nicknam:** Formal analysis (equal); validation (equal). **Atena Bagan:** Funding acquisition (equal); resources (equal); validation (equal). **Solat Eslami:** Formal analysis (equal); methodology (equal); validation (equal). **Azadeh Rakhshan:** Funding acquisition (equal); investigation (equal); visualization (equal). **Soudeh Ghafouri‐Fard:** Project administration (equal); writing – original draft (equal); writing – review and editing (equal).

## CONFLICT OF INTEREST STATEMENT

The authors declare they have no conflict of interest.

## ETHICS STATEMENT

All procedures performed were in accordance with the ethical standards of the institutional and/or national research committee and with the 1964 Helsinki declaration and its later amendments. Informed consent forms were obtained from all study participants. The study protocol was approved by the ethical committee of Shahid Beheshti University of Medical Sciences.

## Supporting information


Table S1.

Appendix S1.

## Data Availability

All data generated or analyzed during this study are included in this published article [and its supplementary information files].
